# Supplementary material for: Vaccination with Neospora GRA6 Interrupts the Vertical Transmission and Partially Protects Dams and Offspring against Neospora caninum Infection in Mice
Source: Vaccines (Basel). 2021 Feb 15;9(2):155. doi: 10.3390/vaccines9020155 (PMC7919036; doi:10.3390/vaccines9020155)
Supplement: Supplementary file 1 [file vaccines-09-00155-s001.pdf]

Supplemental information

Materials and methods

Indirect ELISA to detect NcGRA6- specific antibody

The concentrations of recombinant NcGRA6+GST and GST proteins were adjusted to 0.1 µM each in 0.05 M carbonate buffer (pH 9.6), added to the ELISA plate wells, and the plates were incubated overnight at 4°C. The plates were washed twice with washing buffer (0.05% Tween 20 in PBS) and then blocked with PBS containing 3% skimmed milk (PBS-SM) for 1 h at 37°C. The plates were washed twice and 50 µL of the test serum (diluted 1:100 with PBS-SM), was added to duplicate wells. The plates were incubated at 37°C for 1 h. After the plates were washed six times, they were incubated with horseradish peroxidase-conjugated goat anti-mouse IgG1 or IgG2a antibodies (Bethyl Laboratories, Montgomery, TX, USA) diluted 1:4,000 with PBS-SM, at 37°C for 1 h. The plates were washed six times, and 100 µL of substrate solution (0.1 M citric acid, 0.2 M sodium phosphate, 0.003% H_2O_2, 0.3 mg/mL 2,2′-azino-bis[3-ethylbenzothiazoline-6-sulphonic acid]; Sigma) was added to each well. After sample incubation at room temperature in the dark for 1 h, sample absorbance at 415 nm was determined with a plate reader (Corona Microplate Reader MTP-120; Corona, Tokyo, Japan). The ELISA results for NcGRA6 were determined by measuring the mean optical densities after subtracting from those of GST values.

Results

Generation of specific antibody against NcGRA6 in immunized mouse

In this study, we investigated the levels of IgG1 and IgG2a as markers for triggering protective immunity against NcGRA6 in different mouse groups. Concerning IgG1, immunized mice with NcGRA6+GST showed a significantly higher level of anti-NcGRA6 antibodies against PBS or GST-inoculated groups after third immunization (Figure S1). However, no significant differences were observed in the levels of anti-NcGRA6 in all tested groups (PBS, GST and NcGRA6+GST) in the case of IgG2a antibodies. Noteworthy, antigen-specific antibody levels (IgG1 or IgG2a) against NcGRA6 did not show significant differences among PBS or GST inoculated mice. These results suggest that immunization with rNcGRA6 primarily induced Th2 immune responses against *N. caninum* in the mice.

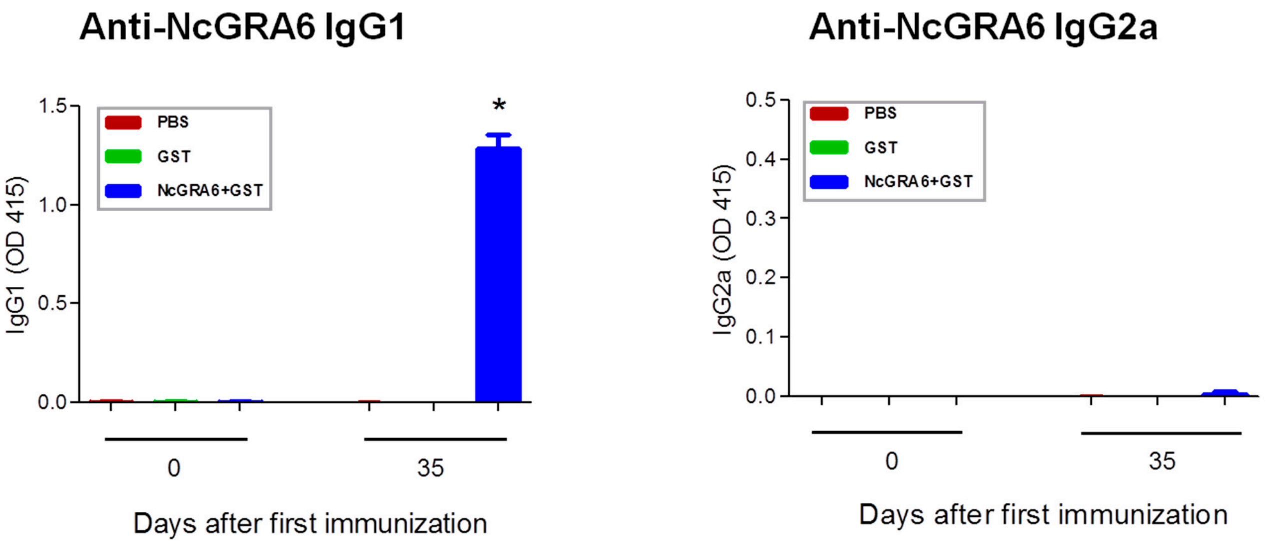

Figure S1. Specific antibody production against NcGRA6 in the immunized and control mice. Sera were collected from three naïve mice (day 0) and from all the mice in each group at day 35 after immunization (PBS = 3, GST = 3, NcGRA6+GST = 4). The antibody responses for each experimental group were tested against recombinant NcGRA6+GST for IgG1 (left panel) and IgG2a (right panel). The data are representative to trial 1 experiment. Each bar represents the mean ± standard

deviation for the mice used per group. *, the statistically significant differences observed for the immunized group against all other groups, one-way ANOVA followed by the Tukey–Kramer, $p < 0.05$.
